# Preliminary Danish Norms for the Odense Child Trauma Screening (OCTS)

**DOI:** 10.1007/s40653-024-00616-7

**Published:** 2024-04-04

**Authors:** Mette Alkærsig, Ask Elklit, Sille Schandorph Løkkegaard

**Affiliations:** 1 The Danish Center of Psychotraumatology, Department of Psychology, University of Southern Denmark, Odense, Denmark; 2 The CH:LD Research Group, Department of Psychology, University of Southern Denmark, Odense, Denmark

**Keywords:** Assessment, Story stem tool, Play, Non-clinical, Narrative, Mental representations

## Abstract

The Odense Child Trauma Screening (OCTS) is Danish story stem screening tool applicable for assessment of preschoolers and young children in risk of being traumatized. Having shown initial evidence of validation, Danish norms are needed to strengthen the clinical assessment with the OCTS by serving as a baseline comparison for assessment of potentially traumatized children. We tested 169 children from the Danish general population aged 4–8 with the OCTS and investigated sex and age differences in play-based behavior and narrative representations. Caregivers reported electronically on child demographic information, psychosocial functioning, and history of trauma exposure using *The Strengths and Difficulties Questionnaire (SDQ)* and *The Diagnostic Infant and Preschool Assessment (DIPA)* trauma list. Across the 145 scores of the OCTS coding scheme, significant sex and age differences were only found in five and sixteen scores respectively. In the five codes where significant sex differences were observed, boys’ normative scores were higher. No significant sex differences were found in the partial story scores or the OCTS total score. Three significant age differences in partial story and OCTS total scores emerged with 4-year-olds scoring higher than 6–8-year-olds. We further found 13 significant age differences in code scores with higher scores among the youngest of the two groups in question suggesting that scores tend to decrease along older age. Few significant sex and age differences were found in children’s OCTS play-based behavior and narrative representations. Indicative of few sex and age biases of the OCTS coding scheme and stories, results suggest that the OCTS can be applied across the intended target group of children aged 4 to 8 years. As higher scores were found in the younger age groups, clinicians should be attentive to age in certain codes of the OCTS coding scheme in their assessment of children in clinical practice. The preliminary normative scores must be interpreted and clinically applied with caution due to our non-representative sample and lack of analyses on factors potentially influencing children’s responses to the OCTS (e.g., developmental, contextual, cultural factors).

**Supplementary Information:**

The online version contains supplementary material available at 10.1007/s40653-024-00616-7.

The Odense Child Trauma Screening (OCTS) is a story stem screening tool developed to, within a structured and controlled setting, screen for play- and narrative-based indications of traumatization in young children (Løkkegaard et al., 2017). Within this frame, "traumatization" is conceptualized as the play-based presentation of psychological difficulties following trauma exposure (Løkkegaard et al., 2017, 2018, 2021). Norms of typical play and narratives among boys and girls at different ages are needed to serve as a baseline of how children from general populations play within the structured and controlled OCTS play-setting. This can strengthen clinicians’ work and reduce the risk of inaccurate or erroneous assessment. Therefore, this study set out to collect preliminary Danish norms for the OCTS and examine potential sex and age differences in play and narratives.

## The Story Stem Tradition

The story stem tradition is a narrative assessment method in which play observation and interview is combined (Bettmann & Lundahl, 2007). The method makes use of symbolic play, storytelling, dolls, or animal figures and is a developmentally sensitive assessment method widely applied with preschoolers and young children (Tang et al., 2018; Steele, 2013; Bettmann & Lundahl, 2007). Through story stems, clinicians can gain insight into children’s mental representations (i.e., internal working models (Bowlby, 1982)) and their strategies for emotion regulation and problem-solving (Emde, 2003). The children are asked to produce a narrative and play-based solution to stories (“stems”) containing familiar external or internal conflicts from everyday life e.g., brief separation from caregivers or waking up in the middle of a night with a nightmare. The themes of the stories are designed to induce a controlled degree of emotional arousal in the child (Plokar & Bassaillon, 2016; Steele, 2013; Bettmann & Lundahl, 2007) because in such a state, the child will draw upon existing mental representations of important others, the world, and the self to create possible solutions to the presented conflict (Emde, 2003; Bretherton et al., 1990d). As such, the play and storytelling will reveal aspects of the ways in which the child tries to make sense of an emotionally unresolved situation (Emde, 2003; Steele, 2013). Many different story stem tools have been developed e.g., Attachment Doll-Play Interview (ADI: Oppenheim, 1997), Attachment Story Completion Task (ASCT: Bretherton et al., 1990c), MacArthur Story Stem Battery (MSSB: Bretherton & Oppenheim, 2003; Bretherton et al., 1990a, 1990b), Manchester Child Attachment Story Task (MCAST: Green et al., 2000), Separation Anxiety Task (SAT: Klagsbrun & Bowlby, 1976), Story Stem Assessment Profile (SSAP: Hodges et al., 2003a, 2004, 2009), and Odense Child Trauma Screening (OCTS: Løkkegaard et al., 2018, 2021). By means of such measures, young children’s voices are amplified as they can become informants in their assessment. Further, they allow for clinicians to systematically collect, deduce, and psychometrically evaluate information about the representational and affective worlds of preschool and young children (Emde, 2003).

### The Pull Factor of Story Stems

What sets story stem tools apart from other narrative methods is the arousal inducing elements, or psychological distress, included in the stories (Emde, 2003). The amount of psychological pressure varies between stems depending on the character of the conflict (e.g., Green et al., 2000; Løkkegaard et al., 2017). The psychological pressure of the stems activates the child’s needs, emotions, and attachment representations and thereby not only invites the child to engage in the story but rather pulls the child into initiating narrative action. By playing out possible solutions to the conflict, the child will attempt to relieve the internal and external psychological pressure induced by the story (Emde, 2003; Green et al., 2003; Løkkegaard et al., 2019). Through different coding systems with specific themes of interest (Bettmann & Lundahl, 2007; Tang et al., 2018), story stem tools can therefore be said to measure each child’s narrative and play-based response to the pull factor (i.e., central conflict).

The central conflicts of the stems are intended to pull in every child interviewee, but it does so to varying degrees. Similar to the various types of responses different cards can elicit in other projective tests (Petersen, & Schilling, 1983; Ridenour et al., 2021), the pull-factor of stems will presumably have a stronger pull on some children and affect other children less intensely. Compared to children from risk groups (e.g., clinical populations, trauma-exposed children), the pull factor will most likely have less of an effect on children from low-risk, non-clinical, or general populations. This mechanism might be understood through theories of emotion regulation since previous studies have found emotion dysregulation (Stargel et al., 2022) or lower emotion regulation competencies in trauma-exposed children compared to non-clinical or control groups (e.g., Langevin et al., 2020; Amédée et al., 2019; Séguin-Lemire et al., 2017; Langevin et al., 2016; Langevin et al., 2015; Shipman et al., 2000). The emotional distressing parts of the story stems might therefore affect risk or clinical groups of children more intensely since their strategies for affect modulation may be less efficient. The varying effect of the pull factor might also be understood through differences in how clinical and children from general populations or non-clinical groups experience and perceive the conflicts of the story stems. Clinical groups of children and/or traumatized children may experience and perceive the distressing parts of the stems as more threatening than their peers from general populations causing higher arousal levels and consequently production of distinct narrative content (Dodd et al., 2012; Steele, 2013). This may cause children from general populations to exert play-based behavior and produce narrative responses that are qualitatively different from those of clinical or risk groups of children.

### Odense Child Trauma Screening (OCTS)

The OCTS includes the following stories: 1) a baseline Birthday stem (contains no central conflict, included to familiarize the child with the test set-up and procedures), 2) a Bike stem (the protagonist falls over on his/her bike) 3) a Nightmare stem, 4) a Burned hand stem, 5) a Stomachache stem, and an optional 6) Animal stem (a little pig gets lost from the rest of the pig family). For clinical use, it is up to the clinician when in the sequence of story stem to apply the animal story stem if deemed needed (Løkkegaard et al., 2017). In the present study, the animal story stem was predominantly introduced as a 6th story stem.

**Fig. 1:**
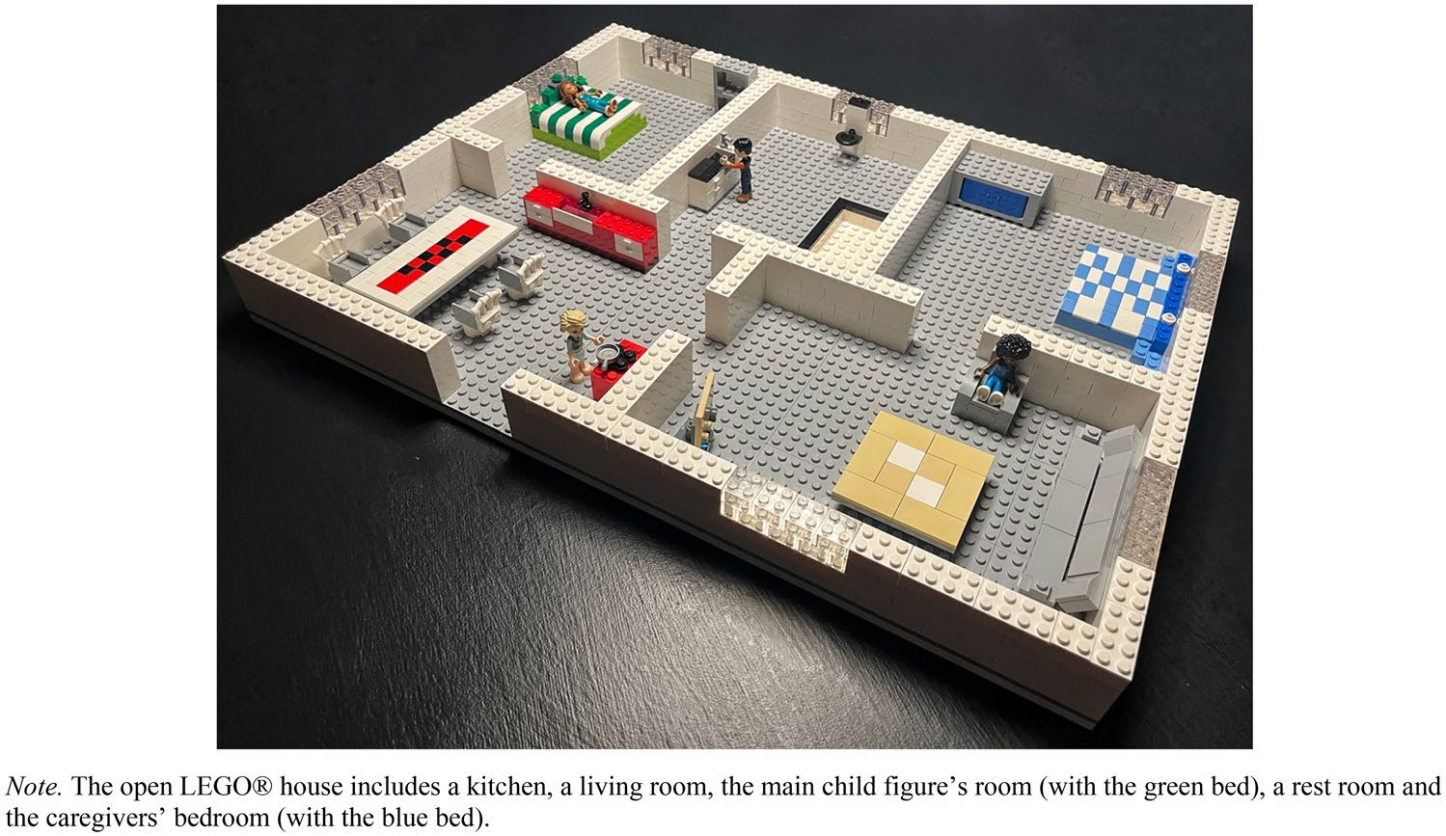
The OCTS test material

The OCTS makes use of LEGO^®^ figures and an open LEGO^®^ house (test material displayed in Fig. 1). Full visibility of the child’s play content and maneuvering of figures is crucial for the subsequent coding of the test and the open house thus ensures higher coding accuracy. Compared to other existing story stem tools, the OCTS contains a higher degree of structure and standardization in both administration and scoring. Total administration time of the OCTS is approximately 30 min and the OCTS can thus be administrated and coded in one and half to two hours (Løkkegaard et al., 2017). For more details on the OCTS and its development, please see Løkkegaard et al., 2021.

### Initial Validation of the OCTS

The OCTS was first tested in a pilot study with 20 children from the general Danish population (Andersen et al., 2016; Løkkegaard, 2019) and has since shown initial evidence of validation with a risk and a community sample (Løkkegaard et al., 2021). Almost half of the children from the risk sample displayed symptoms consistent with the diagnosis of posttraumatic stress disorder (PTSD) or major depression disorder (MDD). None of the children from the community sample displayed symptoms of PTSD or MDD. For the partial score for each story, the internal consistency was good (α = 0.79-0.86) whereas the OCTS total score demonstrated excellent internal consistency (α = 0.95). Initial validation for the OCTS as a screening measure of child traumatization was provided by comparing scores of the OCTS to scores on the Diagnostic Infant and Preschool Assessment (DIPA: Sheeringa & Haslett, 2010) scales of PTSD, MDD and reactive attachment disorder (RAD) and scales of the Strengths and Difficulties Questionnaire (SDQ: Goodman, 1997). The OCTS total score was positively and significantly correlated with the DIPA Total PTSD scale, subscales of re-experiencing and hyperarousal, the MDD scale, and the RAD total scale. The OCTS total score was also positively and significantly correlated with SDQ Total Difficulties scale and three SDQ subscales (conduct problems, hyperactivity, and peer problems). Associations between OCTS total score and DIPA PTSD scale and SDQ Total Difficulties scale were moderate.

### Norms Within Story Stem-based Psychological Assessment

For any given psychological measurement, norms function as common ground for empirical comparisons entailing a higher degree of psychometric accuracy in the interpretation and scoring of a test (Mitrushina et al., 2005). This also holds true for screenings with the OCTS and other story stem-based assessment. In story stem assessment of clinical groups, the test results often function as the corner stone of the succeeding clinical initiatives (e.g., recommendation or planning of further screening, treatment), and it is therefore of utmost importance that the assessment be as accurate, valid, and reliable as possible. This is partly ensured by using standardized and validated story stem tools and by having access to a reference group (i.e., norms) for which to compare the individual test score to.

Despite the vast amount of published literature within the story stem field and the widespread use of story stem tools in assessment, no published study has ever, to our knowledge, generated normative data for a story stem tool. Further, no story stem study has reported on potential sex and age differences in play-based behavior and narrative representations in a large, diverse group of children across all codes of a coding system i.e., all codes, partial, and total scores. Sex and age differences have previously been studied in story stem-contexts among children from low-risk, non-clinical or general populations e.g., with regards to differences in broader concepts such as attachment classification, narrative representations, certain play themes or content and narrative performance (e.g., Vanwalleghem et al., 2021; Kallitsoglou & Repana, 2021; Langevin et al., 2020; Parry et al., 2020; Nóblega et al., 2019; Shin, 2019; Ahmetoglu et al., 2018; Charest et al., 2018; Jin et al., 2018; Gloger-Tippelt & Kappler, 2016). Thus, studies have often reported on sex and age differences in subscales or total scores of a coding scheme. With few exceptions (e.g., Pierrehumbert et al., 2009; von Klitzing et al., 2007), differences have primarily been investigated in studies, including but not limited to the above referenced, where non-clinical/low-risk/typically developing children have been included to function as control groups for clinical groups (e.g., Gregersen et al., 2023; Vanwalleghem et al., 2021; Langevin et al., 2020; Charest et al., 2018; Jin et al., 2018; Gloger-Tippelt & Kappler, 2016). Findings from previous studies have often been contradictory or challenging to draw general conclusions from e.g., due to a) utilization of different story stem tools b) ambiguity with regards to the coding scheme used c) insufficient amounts of information reported on the non-clinical part of the sample d) different statistical handling of data and/or lack of statistical transparency on data from the non-clinical groups (e.g., sex and age sometimes treated as control variables, other times investigated through non-parametric or parametric tests, sometimes statistical handling of data not reported). Consequently, we currently have some knowledge about the play-based behavior and narrative representations of some clinical groups of children (e.g., maltreated and/or traumatized children) in story stem-settings (e.g., Løkkegaard et al., 2021; Charest et al., 2018; Fresno et al., 2018; Beaudoin et al., 2013; Toth et al., 1997; Steele, 2013; Hodges et al., 2003b; Toth et al., 2000; Toth et al., 1997) and little fine-grained data on the story stem participation among diverse groups. Accordingly, as long as norms have not been established for a given story stem tool, psychometric insecurity and a risk of inaccuracy will be embedded into the assessment.

### Study Aim

The aim of this study was therefore to establish preliminary Danish normative scores for the OCTS from a diverse group of children aged 4–8 years from the general Danish population. Normative scores were established for the entire coding scheme (i.e., all codes, partial and total scores). Specific objectives were:

- To document children’s play-based behavior and narrative representations during OCTS and investigate differences depending on sex and age.
- To examine inter-rater coding reliability using intra-class correlation analysis.
- To explore possible associations between scores on the OCTS, scores on the Strengths and Difficulties Questionnaire, and history of trauma exposure.

Documentation and knowledge of the typical play-behavior and narrative representations of children from the general population during OCTS is important for better and more secure identification of deviating responses, behavior, and representations raising cause for concerns of the psychological well-being of a child. We therefore expect that the provision of fine-grained normative scores extracted from a large diverse group of children will contribute to strengthened clinical assessment with the OCTS by 1) reducing the amount of psychometric insecurity embedded in the OCTS assessment 2) enabling clinicians to infer empirically based comparisons when interpreting and scoring individual children’s OCTS test results 3) offering important nuance into the interpretation of ambiguous OCTS test results. Taken together, generating preliminary norms can provide researchers and clinicians a more solid ground from which to interpret OCTS test results and may increase the likelihood that further clinical efforts, such as support or intervention, are initiated for children in need of such initiatives.

## Methods

### Study Design and Approvals

The study was an explorative cross-sectional study approved by SDU Research and Innovation Organization (SDU RIO: #11.512) and by SDU Research Ethics Committee (SDU REC: #21/61473).

### Participants

Children were eligible to participate in the study if they were between 4 years and 0 months and 8 years and 11 months. Children also had to speak Danish at a functional level to participate since the story stem method requires the child to both understand the presented stories and to continue them by narrating themselves. Aiming to secure adequate statistical power to detect between-group differences, we intended to recruit 200 children divided evenly between sex and age levels (with 20 girls and boys from each age level). Following recommendations from Field (2018) as well as guidelines from Ghasemi and Zahediasl (2012) and Pallant (2020), a minimum of n > 30 or 40 participants in each sex and age subgroup would suffice. No special screening for risk status was carried out prior to inclusion of participants since we aimed to recruit a diverse group of children. Children were recruited and tested between January and October 2022 during the COVID-19 pandemic.

Sample demographic characteristics and history of trauma exposure are displayed in Table 1 and 2 respectively. As evident in Table 2, trauma exposure of the current sample was notably lower for most trauma types compared to an included comparison group (the risk group from the initial OCTS validation study: Løkkegaard et al., 2021).

**Table 1:** Demographic sample characteristics (N = 169)*

	**n (%)** **M ± SD**
**Sex (N = 169)**	
Girl	87 (51.5)
Boy	82 (48.5)
**Age (N = 169)**	5.4 ± 1.30
4 years	52 (30.8)
5 years	59 (34.9)
6 years	21 (12.4)
7 years	20 (11.8)
8 years	17 (10.1)
Institution (N = 169)**	
Kindergarten/preschool	119 (70.4)
School	50 (29.6)
**Child’s birth country (N = 120)**	
Denmark Other	119 (99.2) 1 (0.8)
**Biological siblings and half-siblings (N = 108)**	
Min–max	0–6
M ± SD	1.26 ± 0.9
**Caregiver constellation (N = 120)**	
Mother and father	113 (94.2)
Other	2 (1.7)
Not stated	5 (4.2)
**Caregivers’ relationship status (N = 120)**	
Partners/married	100 (83.3)
Separated/divorced	20 (16.7)
**Child’s age at parental separation or divorce (N = 20)**	
Range	0 to + 6 years
Median	2.5
**Country of birth, caregiver(s) (N = 107)**	
Caregiver(s) born in Denmark	93 (86.9)
One or more caregivers born outside of Denmark	14 (13.1)

*M* = mean, *N* = number, *SD* = standard deviation

^*^The reported demographical information is based on the available online caregiver reports. N varies because not all parents who signed their child/children up for participation responded to the questionnaire. Full reports, N = 118

^**^ Of the participating children aged 6, n = 12 were from schools and n = 9 were from kindergarten

**Table 2:** Child experiences of trauma from the DIPA trauma list (N = 117)

Trauma exposure	Caregiver reports N = 117 n (%)	Comparison group N = 31 n (%)
No	72 (61.5)	n/a
Yes	45 (38.5)	31 (100)
Number of traumas experienced, M(SD)	0.76 (1.39)	2.77 (1.2)
**Trauma type**		
Traffic accident (n = 118)	4 (3.4)	0 (0.0)
Attacked by animal	8 (6.8)	1 (3.2)
Natural disasters (hurricane, tornado, flood, etc.)	2 (1.7)	1 (3.2)
Witnessed violence	0 (0.0)	16 (51.6)
Physical abuse	2 (1.7)	18 (58.1)
Sexual abuse	0 (0.0)	10 (32.3)
Accidental burning	10 (8.5)	4 (12.9)
Near drowning	2 (1.7)	2 (3.2)
Hospitalization or invasive medical procedures	20 (17.1)	14 (45.2)
Kidnapped	0 (0.0)	0 (0.0)
Other*	20 (17.1)	19 (61.3)

*M* = mean, *SD* = standard deviation, *DIPA* = Diagnostic Infant and Preschool Assessment, n/a = not applicable

Reports on trauma exposure are missing for 52 of the children included in the study

Comparison group constituted by the risk sample (N = 31) from OCTS validation study (Løkkegaard et al., 2021)

^*^In our sample, the category of “Other” included reports of being born prematurely, choking, resuscitation, parental work-related accidents, general anesthesia, being physically fixated/held down by physicians in relation to physical examinations or medications, a range of physical injuries, divorce, critical illness of a parent or sibling, stillborn siblings, and loss of a loved one

### Measurements

#### Odense Child Trauma Screening (OCTS)

The OCTS is administrated by an interviewer who starts out by narrating and playing out the story stems. With the purpose of engaging the child interviewee in the story and inducing arousal, the administrator narrates the story stems and their distressing parts expressively. After introducing the central conflict, the administrator stops and asks the child to continue the story by “showing and telling what happens next”. The family figures used in the OCTS comprises a mother, a father, a main child figure (“the protagonist”), and a sibling. The protagonist and the sibling figure must match the sex of the child interviewee. To ensure a certain degree of psychological displacement between the experiences of the protagonist and the child interviewee, the name of the protagonist, chosen by the child interviewee, must differ from the child’s own (Løkkegaard et al., 2017).

The OCTS is filmed, transcribed verbatim and subsequently coded based on the video material. The coding scheme contains 27 empirically derived codes divided into five categories: “Engagement and narrative production”, “Nature of the narrative” (coherence and length of the produced narrative), “Adult representations”, “Child representations” and “Disorganized phenomenon”. The OCTS coding scheme is available in the Online Resource 1.

Codes can be assigned the raw scores 0, 1, or 2 reflecting a graduation from “not present” to “definitely present” of the behavior or phenomenon described in each code. Raw scores are recoded into weighted scores of 0 or 1 and are weighed depending on the psychological phenomenon they have been found to be empirically related to. Representations primarily seen in relation to traumatization are thus weighed differently (i.e., raw score of either 1 or 2 recoded into a weighted score of 1) than representations seen in children with other clinically symptomatic behavior (i.e., only raw scores of 2 are recoded into a weighted score of 1).

By summing up weighted scores, a partial score is calculated for every conflict stem. A total score is calculated by first summing up the partial scores for each completed conflict stem and afterwards dividing the sum by the number of completed conflict stems. A high OCTS total score indicate that the child screens positive for plausible traumatization (i.e., in their response, the child has expressed themes, play behavior, or narrative representations associated with traumatization or a profound degree of phenomena associated with other vulnerability) and further assessment and differential diagnosis is needed (Løkkegaard et al., 2018). A clinical cut-off score for the OCTS has not yet been possible to establish since no other golden standard trauma-focused assessment tool with children as informants exists for the age group. For a more detailed description of the development of the coding system and its empirical underpinnings, see Løkkegaard et al. (2018, 2021). Administration and coding manuals referenced here are the English versions. For the present study, the Danish versions were used.

#### Caregiver-reported Data

Children’s demographic information, history of trauma exposure, and psychosocial functioning was obtained through an online caregiver-reported questionnaire. Caregivers were sent the link to the questionnaire through the website for home-school/kindergarten communication (“intranet”). Reminders to respond was continuously reposted and in several cases sent directly through intranet to caregivers who had not yet responded.

#### Strengths and Difficulties Questionnaire (SDQ)

The SDQ screens for behavioral, emotional, and social difficulties in children between 2 and 17 years of age (Goodman, 1997). For this study, the caregiver report version was used for preschool and school children. The SDQ consists of 25 questions distributed evenly among five subscales covering emotional symptoms, conduct problems, hyperactivity/inattention, peer relationship problems, and prosocial behavior. The SDQ also contains an optional impact supplement which asks as to whether the respondent thinks the child or young person has general difficulties in either one, some, or all the following areas: Concentration, behavior, relations, and emotions. If so, the impact supplement further inquiries about duration of difficulties, whether the difficulties cause the child or young person distress, impairs the person’s learning abilities, or social interactions and whether the difficulties are a burden to others (i.e., the family in the caregiver version). The impact supplement was included in our study.

The psychometric properties of the different versions of the SDQ and its five-factor structure has been examined in several studies. Niclasen and colleagues (2012) investigated the psychometric properties of the SDQ in a sample of 71,840 Danish children in the age groups 5–7 years and 10–12 years. The study confirmed the five-factor structure and generally found high reliability coefficients for the subscales, the Total difficulties scale, and the Impact scale (Cronbach’s alpha ranging between 0.44–0.88). The usefulness of SDQ in the general Danish population was thus supported. Danish norms are also available (Arnfred et al., 2019).

#### Diagnostic Infant and Preschool Assessment (DIPA): Trauma list

The DIPA is a diagnostic caregiver interview for assessment of preschool children (Scheeringa & Haslett, 2010). It covers 13 different psychopathological disorders, one of them being PTSD. The PTSD module includes a trauma list of 11 types of potential traumatic events (PTE) and a category of “other traumas” (see Table 2). The Danish version of the trauma list was included in the online questionnaire and caregivers therefore reported on trauma exposure electronically. Unfortunately, a few months into the data collection, we discovered that we failed to include event 3 on the trauma list “man-made disasters (fires, war etc.)” in the questionnaire. Hence, we cannot report on the prevalence of this trauma type in our sample.

### Procedures

#### Recruitment

**Fig. 2:**
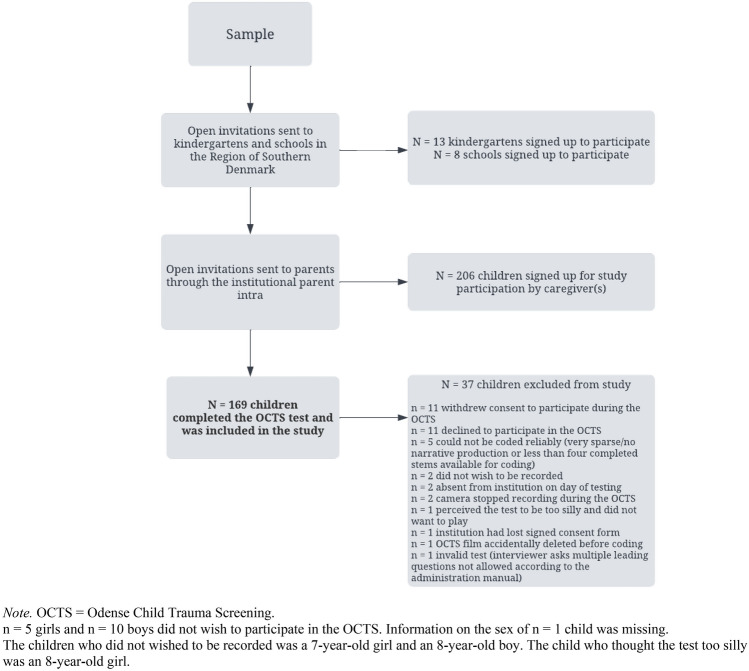
Flow of participant recruitment (N = 169)

Children were recruited from public kindergartens and schools in both urban and rural areas in the Region of Southern Denmark. A few children were recruited from the Capital Region of Denmark. Open study invitations were sent to all public kindergartens and schools on Fuenen with contact information available on their website, to selected institutions in the Capital Region of Denmark, and other parts of the Region of Southern Denmark. Invitations were also distributed through our networks of teachers and psychologists. Reminders to respond to the invitation were sent 1–3 times between 2–4 weeks after the initial invitation. Caregivers received study invitation and information through the institutional electronic site for home-school/kindergarten communication (intranet). Participant recruitment flow is illustrated in Fig. 2. Potential differences between children who completed the OCTS and children who either declined to participate beforehand or during the test were not explored due to the small group sizes (n = 11 and n = 11).

#### OCTS Testing, Test Administrators and Coders

The OCTS tests were conducted during institutional hours in quiet rooms in kindergartens and schools. Present was one test administrator, the child, and occasionally another psychology student contributing to the data collection. In cases where a child felt more comfortable with the presence of a familiar adult, a child’s pedagogue or parent(s) were allowed to observe the OCTS test as well.

Test administrators (n = 12) and coders (n = 16) were all psychology students at the University of Southern Denmark. Eleven coders were enrolled in the master’s program while four were bachelor level students. The first author also functioned as a test administrator. All test administrators and coders had to participate in a two-day training course in the administration and scoring of the OCTS taught by the 3rd author. To ensure reliable administrations, administrators received supervision throughout the data collection. To become reliable coders, coders rehearsed the coding of OCTS with three to five films and received supervision on their coding of these films by the 1st and 3rd author. In complex cases or when in doubt of a specific coding, coders could seek out supervision. All coders were blind to information about study purpose and participating children, and blind double coding was carried out on 25% (n = 43) of the OCTS tests. One coding was a priori assigned as the primary coding and was used for all other analyses other than the interrater reliability analysis. One challenging OCTS test was coded by a third coder (the 3rd author) unfortunately not blind to study aims. Due to inadequate narrative production from the child, this test could not be coded reliably and was deemed invalid. It was excluded from all analyses including inter-rater reliability analyses.

### Ethical Considerations

Active, informed written consent was given by all child caregivers with child custody before the OCTS testing. Children gave oral consent to participate before initiation of the OCTS test. Prior, they were informed of the study in an age-appropriate manner and explained about their right to withdraw consent and terminate participation at any given time during the test.

The emotional distress and arousal induced in children by the OCTS conflict stems may cause children previously exposed to PTE to spontaneously disclose of such experiences (e.g., physical violence, sexual abuse, psychological violence) during the OCTS test. It was decided beforehand that should such a situation occur during the data collection test administrators had to discontinue the test and turn off the video camera recording. We developed a manuscript for test administrators to make use of hereafter. The phrases in the manuscript were developed to help the administrator keep calm and aid them in managing the situation and interact with the child in an ethically responsible, age-appropriate, and gentle manner. In addition, we developed written guidelines of the actions the administrator and research group had to take after a test where a child spontaneously disclosed having been exposed to one or more PTE. Included in this guideline were also guidelines for actions to take in cases where a OCTS test gave rise to either subtle or serious worries about a child’s psychological wellbeing. During the training, the test administrators were thoroughly introduced to the content of both guidelines. In six cases, the 2nd and 3rd authors were involved in the assessment of subtle concerns raised by test administrators. There was no cause for action in any of the six cases.

### Statistics

Descriptive analyses were conducted to establish preliminary normative scores for all codes, partial and total scores. Normative scores were operationalized as mean scores due to the complexity of the OCTS coding scheme. Potential sex and age differences in preliminary norm scores were investigated by use of One-Way Analysis of Variance (ANOVA), as the sample sizes of the subgroups (across sex and age levels) were deemed large enough for parametric analyses (Elliot & Woodward, 2007; Field, 2018; Ghasemi & Zahediasl, 2012). To examine potential associations between scores on the OCTS and SDQ, Spearman’s rho was used as the number of available SDQ-responses for some subgroups were estimated more fit for non-parametric methods (Elliot & Woodward, 2007; Pallant, 2020). Spearman’s rho was also used to assess associations between scores on the OCTS and number of trauma exposure(s). Inter-rater reliability was calculated using intraclass correlation coefficients and internal consistency was assessed using Cronbach’s alpha.

## Results

The final sample comprised 169 children (M = 5.41 years, SD = 1.30), and full online caregiver reports were available for 118 children (Table 1, response rate = 70%). For 154 children, all five narrative responses to conflict stems could be coded reliably, whereas 15 children (9%) produced only four narrative responses that could be coded reliably. In cases where a narrative could not be coded reliably, the partial score was assigned the value 99 and the stem was excluded from all analyses. The remaining four narrative responses with valid partial scores were included. As the information can be of value in clinical contexts, an overview of the incomplete stems and reasons for their invalidity are presented in Table 3.

**Table 3:** List of reasons for the invalidity of incomplete stems (N = 15) in included OCTS-tests

Stem	Reason for invalidity	N
Bike	Child did not engage in narrative production.	1
Stomachache	Child did not engage in narrative production.	2
Stomachache	Child did not want to play out the stem.	2
Animal	Child did not engage in narrative production.	1
Animal	Child did not produce enough narrative content for reliable coding.	1
Animal	Interviewer accidentally described the stomachache stem as the last stem of the test and thus decided not to play out the animal stem.	1
Animal	Interviewer decided not to play out the animal stem due to child’s exhaustion level.	1
Animal	Child did not want to play the story.	1
Animal	Technical issues: The stem was played out but was not recorded.	2
Animal	The interviewer thought the guideline for maximum administration time limit was surpassed and thus decided to skip the animal stem.	3

*N* = number

The calculated normative scores across sex and age will be reported on three levels 1) OCTS total score, 2) OCTS partial scores (story scores), and 3) code scores. As addressed in the methods section, the OCTS coding scheme includes 27 codes nested within five coding categories, all describing certain play-based behavior or narrative representations that might be present or absent during the child’s narrative response to the OCTS stories. As all 27 codes must be assigned a score in the five OCTS conflict stories in addition to the scoring of four codes in the preceding baseline story as well as partial scores and a total score, 145 significant sex or age differences in scores could thus potentially emerge (OCTS coding scheme available in Online Resource 1).

No significant differences were found in OCTS partial or total scores between groups of children with and without divorced caregivers or between groups of children with all caregivers born in Denmark and children with one or more caregivers born outside of Denmark. Therefore, these demographic data were excluded from further analyses.

### Preliminary OCTS Normative Scores and Sex Differences

#### OCTS Partial and Total Scores

**Fig. 3:**
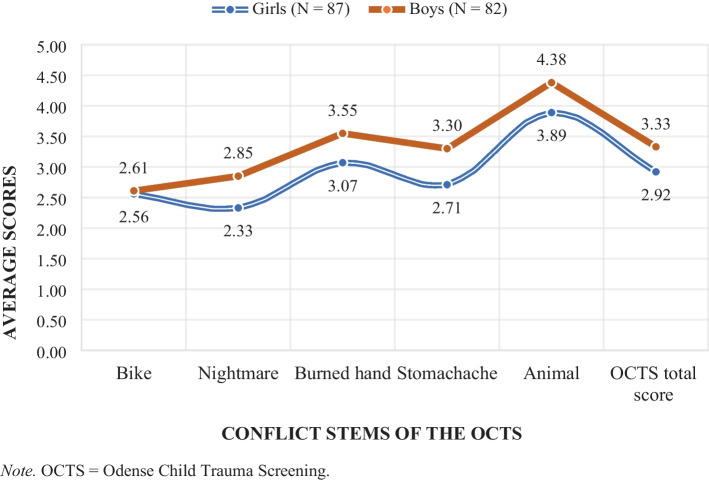
Preliminary normative OCTS partial and total scores aggregated by sex

The normative scores on OCTS codes, partial, and total scores depending on sex are presented in Table 4. Slightly higher average partial and total scores were consistently observed among boys (see Table 4 and Fig. 3). However, none of these sex differences were statistically significant.

**Table 4:** Preliminary normative OCTS scores aggregated by sex

	**Normative scores** **M(SD)**																	
	**Baseline**			**Story stem 1:** **Bike**			**Story stem 2:** **Nightmare**			**Story stem 3:** **Burned** **hand**			**Story stem 4:** **Stomachache**			**Story stem 5:** **Animal**		
	**G**	**B**	**F**	**G**	**B**	**F**	**G**	**B**	**F**	**G**	**B**	**F**	**G**	**B**	**F**	**G**	**B**	**F**
**Engagement and narrative production**																		
**1. Engagement**	0.07 (0.37)	0.02 (0.22)	.902	0.02 (0.21)	0.00 (0.00)	.942	0.02 (0.21)	0.02 (0.22)	.002	0.00 (0.00)	0.00 (0.00)	n/a	0.07 (0.37)	0.02 (0.22)	.902	0.02 (0.22)	0.03 (0.23)	.002
**2. Arousal**				0.02 (0.21)	0.05 (0.31)	.399	0.02 (0.21)	0.02 (0.22)	.002	0.02 (0.21)	0.07 (0.38)	1.110	0.00 (0.00)	0.00 (0.00)	n/a	0.02 (0.22)	0.03 (0.23)	.002
**3. Arousal modulation**				0.02 (0.21)	0.02 (0.22)	.002	0.00 (0.00)	0.02 (0.22)	-	0.02 (0.21)	0.00 (0.00)	.942	0.07 (0.37)	0.02 (0.22)	.902	0.00 (0.00)	0.00 (0.00)	n/a
**4. Ability to keep within task boundaries**	0.05 (0.30)	0.05 (0.31)	.004	0.02 (0.21)	0.05 (0.31)	.399	0.05 (0.30)	0.05 (0.31)	.004	0.03 (0.24)	0.02 (0.22)	.081	0.07 (0.37)	0.05 (0.31)	.148	0.05 (0.31)	0.05 (0.32)	.004
**Nature of the narrative**																		
**5. Narrative coherence**	0.73 (0.83)	0.51 (0.76)	3.356	0.58 (0.77)	0.55 (0.71)	.081	0.59 (0.79)	0.60 (0.73)	.009	0.51 (0.73)	0.51 (0.71)	.003	0.51 (0.69)	0.57 (0.74)	.254	0.61 (0.76)	0.45 (0.66)	2.189
**6. Interviewer support**	0.70 (0.83)	0.55 (0.74)	1.508	0.49 (0.75)	0.39 (0.68)	.789	0.36 (0.63)	0.28 (0.57)	.670	0.41 (0.71)	0.29 (0.53)	1.591	0.40 (0.69)	0.23 (0.57)	3.072	0.23 (0.50)	0.12 (0.40)	2.473
**Adult representations in the narrative**																		
**7. Adult provides comfort**				1.67 (0.69)	1.59 (0.75)	.637	1.57 (0.74)	1.67 (0.63)	.819	1.81 (0.52)	1.78 (0.59)	.175	1.76 (0.59)	1.72 (0.68)	.215	1.84 (0.48)	1.96 (0.25)	**3.934**
**8. Adult provides help/protection**				0.66 (0.80)	0.71 (0.82)	.150	0.60 (0.86)	0.79 (0.91)	2.055	0.85 (0.88)	1.04 (0.86)	1.968	0.86 (0.88)	0.91 (0.90)	.166	1.06 (0.96)	1.12 (0.96)	.135
**9. Adult unaware**				0.35 (0.76)	0.32 (0.73)	.075	0.36 (0.76)	0.33 (0.74)	.055	0.16 (0.55)	0.10 (0.44)	.657	0.36 (0.77)	0.52 (0.87)	1.592	0.51 (0.83)	0.71 (0.94)	2.060
**10. Adult rejects child**				0.03 (0.24)	0.04 (0.19)	.003	0.02 (0.15)	0.09 (0.28)	3.176	0.07 (0.25)	0.10 (0.30)	.450	0.11 (0.41)	0.04 (0.19)	1.999	0.04 (0.19)	0.04 (0.25)	.003
**11. Adult shows aggression**				0.0 (0.00)	0.02 (0.22)	-	0.00 (0.00)	0.02 (0.22)	-	0.02 (0.15)	0.05 (0.31)	.481	0.07 (0.37)	0.07 (0.35)	.002	0.23 (0.61)	0.52 (0.87)	**5.757**
**12. Adult is controlling**				0.02 (0.22)	0.05 (0.22)	.585	0.05 (0.21)	0.01 (0.11)	1.732	0.07 (0.33)	0.23 (1.03)	1.850	0.06 (0.28)	0.00 (0.00)	-	0.00 (0.00)	0.01 (0.11)	-
**Child representations in the narrative**																		
**13. Child seeks help/comfort from adult**				0.40 (0.74)	0.40 (0.77)	.004	0.24 (0.63)	0.34 (0.69)	.976	0.78 (0.95)	1.06 (0.96)	3.655	0.42 (0.71)	0.64 (0.90)	2.990	1.00 (1.00)	0.99 (0.98)	.007
**14. Avoidance of conflict**				0.24 (0.51)	0.23 (0.45)	.028	0.36 (0.61)	0.30 (0.56)	.325	0.37 (0.65)	0.33 (0.52)	.180	0.29 (0.59)	0.21 (0.44)	.877	0.05 (0.27)	0.14 (0.42)	2.733
**15. Child self-care**				0.25 (0.58)	0.30 (0.64)	.376	0.23 (0.54)	0.26 (0.56)	.095	0.48 (0.78)	0.68 (0.90)	2.325	0.37 (0.69)	0.60 (0.83)	**3.914**	0.98 (0.97)	0.99 (0.95)	.006
**16. Child ‘parents’ or ‘controls’ adult**				0.00 (0.00)	0.00 (0.00)	n/a	0.02 (0.21)	0.00 (0.00)	.942	0.00 (0.00)	0.02 (0.16)	-	0.02 (0.22)	0.00 (0.00)	-	0.00 (0.00)	0.00 (0.00)	n/a
**17. Excessive compliance in child**				0.00 (0.00)	0.00 (0.00)	n/a	0.00 (0.00)	0.00 (0.00)	n/a	0.07 (0.37)	0.00 (0.00)	-	0.04 (0.24)	0.02 (0.22)	.092	0.00 (0.00)	0.00 (0.00)	n/a
**18. Child shows aggression**				0.06 (0.32)	0.15 (0.52)	1.713	0.01 (0.11)	0.07 (0.38)	2.137	0.02 (0.21)	0.05 (0.27)	.481	0.05 (0.31)	0.04 (0.19)	.070	0.24 (0.64)	0.19 (0.56)	.263
**19. Child ambivalence**				0.05 (0.21)	0.04 (0.19)	.102	0.03 (0.18)	0.09 (0.36)	1.325	0.05 (0.21)	0.10 (0.37)	1.210	0.06 (0.24)	0.14 (0.44)	1.897	0.02 (0.22)	0.03 (0.16)	.003
**20. Child shows fear of adult**				0.00 (0.00)	0.00 (0.00)	n/a	0.01 (0.11)	0.00 (0.00)	.942	0.00 (0.00)	0.01 (0.11)	-	0.00 (0.00)	0.01 (0.11)	-	0.04 (0.19)	0.10 (0.42)	1.687
**21. Assuagement**				0.58 (0.68)	0.57 (0.69)	.012	0.49 (0.63)	0.63 (0.75)	1.601	0.57 (0.76)	0.62 (0.72)	.172	0.49 (0.61)	0.53 (0.69)	.092	0.41 (0.61)	0.36 (0.58)	.299
**Disorganised phenomena**																		
**22. Bizarre elements in the narrative**				0.15 (0.47)	0.18 (0.52)	.170	0.09 (0.33)	0.28 (0.57)	**6.789**	0.09 (0.33)	0.18 (0.45)	2.245	0.17 (0.46)	0.19 (0.48)	.064	0.18 (0.45)	0.18 (0.48)	.000
**23. Child/adult gets hurt or dies**				0.43 (0.74)	0.35 (0.71)	.465	0.09 (0.33)	0.20 (0.53)	2.273	0.24 (0.57)	0.34 (0.69)	1.052	0.18 (0.54)	0.16 (0.51)	.049	0.60 (0.86)	0.62 (0.87)	.035
**24. Child/adult/things are thrown away**				0.02 (0.22)	0.00 (0.00)	-	0.05 (0.30)	0.00 (0.00)	-	0.05 (0.30)	0.07 (0.38)	.269	0.07 (0.37)	0.02 (0.22)	.946	0.00 (0.00)	0.04 (0.25)	-
**25. Sudden shifts between good and bad**				0.00 (0.00)	0.01 (0.11)	-	0.00 (0.00)	0.02 (0.16)	-	0.00 (0.00)	0.02 (0.22)	-	0.01 (0.11)	0.00 (0.00)	-	0.05 (0.27)	0.05 (0.28)	.005
**26. Sexual material**				0.00 (0.00)	0.00 (0.00)	n/a	0.00 (0.00)	0.00 (0.00)	n/a	0.00 (0.00)	0.00 (0.00)	n/a	0.00 (0.00)	0.00 (0.00)	n/a	0.00 (0.00)	0.01 (0.11)	-
**27. Disorganised behaviour**				0.15 (0.42)	0.16 (0.43)	.013	0.13 (0.37)	0.22 (0.50)	1.896	0.15 (0.42)	0.21 (0.46)	.726	0.13 (0.37)	0.30 (0.60)	**4.470**	0.06 (0.24)	0.16 (0.37)	3.692
**Partial score per story**				2.56 (2.25)	2.61 (2.71)	.018	2.33 (2.08)	2.85 (2.48)	2.195	3.07 (2.38)	3.55 (2.76)	1.470	2.71 (2.27)	3.30 (2.46)	2.502	3.89 (2.26)	4.38 (2.54)	1.631

				**Girls** (N = 87)						**Boys** (N = 82)						**One-way ANOVA**		
**OCTS total score** **M(SD)** **Min–max**				2.92 (1.61) 0.20–7.60						3.33 (1.91) 0.60–9.80						F(1, 167) = 2.240, p = .136		

*ANOVA* = Analysis of variance, *B* = Boys, *F* = F-value, One-way ANOVA, *G* = Girls, *M* = mean, *Min* = minimum score observed, *Max* = maximum score observed, n/a = all absolute deviations are constant within each cell, albeit both groups (boys and girls) scored 0 in the code in question, SD = standard deviation

Descriptive analyses on mean code scores and partial scores were based on N = 79–87 scores for girls and N = 75–82 scores for boys

Degrees of freedom for all ANOVA analyses were set to 1

The significance level is p < .05. Statistically significant sex differences are highlighted in bold. None were statistically significant at the level of p < .01

- = robust test of equality means analyses could not be performed because at least one group had 0 variance

Robust test of equality of means (Brown-Forsythe) were performed in the following codes and stems: 2 (Burned Hand), 5 (Animal), 6 (Stomachache, Animal), 7 (Animal), 9 (Stomachache, Animal), 10 (Nightmare, Stomachache), 12 (Nightmare, Burned Hand), 13 (Stomachache, Animal), 14 (Stomachache, Animal), 15 (Burned Hand, Stomachache), 18 (Bike, Nightmare), 19 (Nightmare, Burned Hand, Stomachache), 20 (Animal), 21 (Nightmare), 22 (Nightmare, Burned Hand), 23 (Nightmare, Burned Hand), 27 (Nightmare, Stomachache, Animal)

#### Code scores

Across the 139 codes of the OCTS baseline and conflict stems, statistically significant sex differences were only found in five codes. In all five cases, boys consistently scored significantly higher than girls. Significantly more bizarre elements were present in the narratives of boys in the Nightmare stem (code 22: F(1, 127.439) = 6.789, p = 0.010), the child protagonist in boys’ narratives displayed significantly more self-help in relation to the central conflict in the Stomachache stem (code 15: F(1, 155.460) = 3.914, p = 0.050) and the narratives of boys contained significantly more disorganization in the Stomachache stem (code 27: F(1, 132.858) = 4.470, p = 0.036). Boys’ adult representations were also displayed as significantly more aggressive (code 11: F(1, 136.130) = 5.757, p = 0.018) and significantly less comforting (code 7: F(1, 123.248) = 3.934, p = 0.050) in the Animal stem compared to those of girls. The sex differences in the following five normative code scores were close to reaching statistical significance: Code 5 (F(1, 165) = 3.356, p = 0.069) with poorer narrative coherence in girls’ baseline narratives, Nightmare, code 10 (F(1, 122.376) = 3.176, p = 0.077) with more rejecting adult representations among boys, Burned hand, code 13 (F(1, 166) = 3.655, p = 0.058) with child representations as less help-seeking in narrative responses of boys, Stomachache, code 6 (F(1, 164.442) = 3.072, p = 0.082) with girls needing more interviewer support in this stem, Animal, code 27 (F(1, 130.350) = 3–692, p = 0.057) with more disorganized phenomena in the narratives of boys.

In 13 code scores across the coding scheme, no variance was observed (all children scored 0, see Table 4) and analyses on differences in normative scores were thus not calculated. For scores on 18 codes, robust tests of equality of means (Brown-Forsythe) could not be performed because one of the groups had no variance in mean scores (all children in one of the groups scored 0, see Table 4: Bike scores, codes: 11, 24, 25. Nightmare scores, codes: 3, 11, 24, 25. Burned hand scores, codes: 16, 17, 20, 25, Stomachache scores, codes: 12, 16, 20, 25. Animal scores, codes: 12, 24, 26). Potential sex differences were not calculated for these norm scores either.

### Preliminary OCTS Normative Scores and Age Differences

#### OCTS Partial and Total Scores

**Fig. 4:**
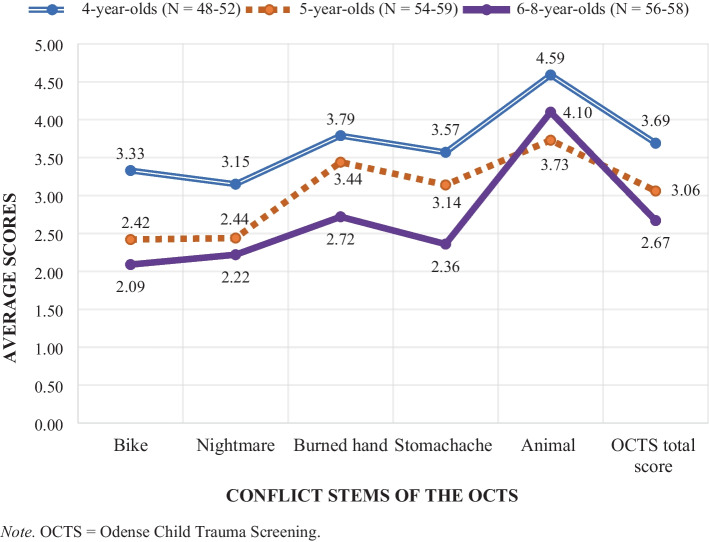
Preliminary normative OCTS partial and total scores aggregated by age

Age-aggregated code, partial, and total normative scores are presented in Table 5. OCTS partial and total scores are illustrated in Fig. 4. Children aged 6–8 were clustered together as fewer children were recruited across these ages resulting in too small subgroup sizes for independent parametric examination (Ghasemi & Zahediasl, 2012). Across all age groups, significant age differences in the normative partial and total scores were only detected between the age groups of 4 and 6–8 years. Significant differences were only found in the partial scores of the Bike stem (F(2, 165) = 3.755, p = 0.025, Tukey HSD post-hoc analysis, p = 0.023) and Stomachache stem (F(2, 162) = 3.784, p = 0.025, Tukey HSD post-hoc analysis, p = 0.021) and in OCTS total scores (F(2, 166) = 4.901, p = 0.009, Tukey HSD post-hoc analysis, p = 0.006) with higher scores in the group of 4-year-olds.

**Table 5:** Preliminary normative OCTS scores aggregated by age

	**Normative scores** **M(SD)**																	
	**Baseline**			**Story stem 1:** **Bike**			**Story stem 2:** **Nightmare**			**Story stem 3:** **Burned** **hand**			**Story stem 4:** **Stomachache**			**Story stem 5:** **Animal**		
	**4 yrs**	**5 yrs**	**6–8 yrs**	**4 yrs**	**5 yrs**	**6–8 yrs**	**4 yrs**	**5 yrs**	**6–8 yrs**	**4 yrs**	**5 yrs**	**6–8 yrs**	**4 yrs**	**5 yrs**	**6–8 yrs**	**4 yrs**	**5 yrs**	**6–8 yrs**
**1. Engagement**	0.12 (0.47)	0.00 (0.00)	0.03 (0.26)	0.04 (0.28)	0.00 (0.00)	0.00 (0.00)	0.04 (0.28)	0.00 (0.00)	0.03 (0.26)	0.00 (0.00)	0.00 (0.00)	0.00 (0.00)	0.04 (0.28)	0.10 (0.44)	0.00 (0.00)	0.04 (0.28)	0.04 (0.26)	0.00 (0.00)
**2. Arousal**				0.00 (0.00)	0.00 (0.00)	0.10 (0.45)	0.00 (0.00)	0.00 (0.00)	0.07 (0.37)	0.04 (0.28)	0.00 (0.00)	0.10 (0.45)	0.00 (0.00)	0.00 (0.00)	0.00 (0.00)	0.04 (0.28)	0.00 (0.00)	0.04 (0.28)
**3. Arousal modulation**				0.04 (0.28)	0.03 (0.26)	0.00 (0.00)	0.04 (0.28)	0.00 (0.00)	0.00 (0.00)	0.04 (0.28)	0.00 (0.00)	0.00 (0.00)	0.08 (0.39)	0.07 (0.37)	0.00 (0.00)	0.00 (0.00)	0.0 (0.00)	0.0 (0.00)
**4. Ability to keep within task boundaries**	0.12 (0.47)	0.03 (0.26)	0.00 (0.00)	0.00 (0.00)	0.07 (0.37)	0.03 (0.26)	0.12 (0.47)	0.03 (0.26)	0.00 (0.00)	0.04 (0.28)	0.03 (0.26)	0.02 (0.13)	0.08 (0.39)	0.07 (0.37)	0.03 (0.26)	0.12 (0.47)	0.04 (0.26)	0.0 (0.00)
**Nature of the narrative**																		
**5. Narrative coherence**	0.72 (0.83)	0.71 (0.85)	0.45 (0.71)	0.65 (0.74)	0.54 (0.77)	0.52 (0.71)	0.73 (0.79)	0.56 (0.77)	0.50 (0.71)	0.62 (0.77)	0.59 (0.75)	0.33 (0.60)	0.51 (0.70)	0.70 (0.78)	0.41 (0.62)	0.54 (0.70)	0.60 (0.75)	0.46 (0.70)
**6. Interviewer support**	0.73 (0.85)	0.73 (0.83)	0.43 (0.65)	0.53 (0.76)	0.53 (0.77)	0.28 (0.59)	0.37 (0.66)	0.37 (0.61)	0.22 (0.53)	0.44 (0.73)	**0.44** **(0.68)**	**0.19** **(0.44)**	0.38 (0.72)	0.42 (0.70)	0.16 (0.45)	0.22 (0.46)	0.25 (0.58)	0.06 (0.24)
**Adult representations in the narrative**																		
**7. Adult provides comfort**				1.67 (0.68)	**1.78** **(0.59)**	**1.45** **(0.84)**	1.67 (0.65)	1.69 (0.59)	1.50 (0.80)	1.75 (0.62)	1.88 (0.46)	1.76 (0.57)	1.82 (0.56)	**1.86** **(0.48)**	**1.55** **(0.78)**	1.92 (0.33)	1.86 (0.48)	1.92 (0.33)
**8. Adult provides help/protection**				**0.94** **(0.88)**	0.67 (0.78)	**0.47** **(0.71)**	0.77 (0.92)	0.64 (0.91)	0.67 (0.85)	1.12 (0.92)	0.91 (0.93)	0.81 (0.76)	1.12 (0.91)	0.80 (0.88)	0.76 (0.84)	**1.40** **(0.87)**	**0.93** **(0.97)**	0.96 (0.97)
**9. Adult unaware**				0.43 (0.83)	0.37 (0.79)	0.21 (0.61)	0.46 (0.85)	0.36 (0.76)	0.22 (0.62)	0.08 (0.39)	**0.28** **(0.70)**	**0.03** **(0.26)**	**0.65** **(0.93)**	0.44 (0.83)	**0.24** **(0.66)**	0.59 (0.89)	0.42 (0.79)	0.82 (0.95)
**10. Adult rejects child**				0.06 (0.24)	0.00 (0.00)	0.05 (0.29)	0.06 (0.24)	0.00 (0.00)	0.10 (0.31)	0.08 (0.27)	0.08 (0.28)	0.09 (0.28)	0.10 (0.41)	0.02 (0.13)	0.10 (0.36)	0.02 (0.14)	0.04 (0.19)	0.06 (0.31)
**11. Adult shows aggression**				0.04 (0.28)	0.00 (0.00)	0.00 (0.00)	0.00 (0.00)	0.03 (0.26)	0.00 (0.00)	0.08 (0.39)	0.00 (0.00)	0.03 (0.18)	0.04 (0.28)	0.05 (0.30)	0.12 (0.46)	0.20 (0.57)	0.41 (0.78)	0.50 (0.87)
**12. Adult is controlling**				0.06 (0.31)	0.02 (0.13)	0.03 (0.18)	0.02 (0.14)	0.03 (0.18)	0.03 (0.18)	0.29 (1.29)	0.08 (0.28)	0.09 (0.34)	0.00 (0.00)	0.02 (0.13)	0.07 (0.32)	0.00 (0.00)	0.02 (0.13)	0.00 (0.00)
**Child representations in the narrative**																		
13. Child seeks help/comfort from adult*				0.49 (0.81)	0.44 (0.77)	0.28 (0.67)	0.42 (0.75)	0.22 (0.59)	0.24 (0.63)	1.08 (0.98)	0.93 (0.98)	0.76 (0.92)	0.69 (0.88)	0.57 (0.85)	0.34 (0.69)	**1.35** **(0.93)**	**0.82** **(0.97)**	**0.83** **(0.98)**
**14. Avoidance of conflict**				0.35 (0.59)	0.24 (0.47)	0.14 (0.35)	0.52 (0.73)	0.25 (0.51)	0.24 (0.47)	**0.52** **(0.73)**	0.36 (0.58)	**0.19** **(0.40)**	0.35 (0.63)	0.29 (0.53)	0.12 (0.38)	0.17 (0.47)	0.07 (0.32)	0.04 (0.19)
**15. Child self-care**				0.39 (0.70)	0.24 (0.57)	0.21 (0.55)	0.25 (0.56)	0.27 (0.61)	0.21 (0.49)	0.56 (0.86)	0.55 (0.89)	0.62 (0.79)	0.65 (0.87)	0.44 (0.74)	0.38 (0.70)	**1.27** **(0.92)**	**0.77** **(0.97)**	0.92 (0.93)
**16. Child ‘parents’ or ‘controls’ adult**				0.00 (0.00)	0.00 (0.00)	0.00 (0.00)	0.00 (0.00)	0.00 (0.00)	0.03 (0.26)	0.00 (0.00)	0.02 (0.13)	0.02 (0.13)	0.00 (0.00)	0.00 (0.00)	0.03 (0.26)	0.00 (0.00)	0.00 (0.00)	0.00 (0.00)
**17. Excessive compliance in child**				0.00 (0.00)	0.00 (0.00)	0.00 (0.00)	0.00 (0.00)	0.00 (0.00)	0.00 (0.00)	0.04 (0.28)	0.03 (0.26)	0.03 (0.26)	0.00 (0.00)	0.05 (0.30)	0.03 (0.26)	0.00 (0.00)	0.00 (0.00)	0.00 (0.00)
**18. Child shows aggression**				0.08 (0.39)	0.08 (0.38)	0.14 (0.51)	0.00 (0.00)	0.03 (0.26)	0.09 (0.39)	0.04 (0.28)	0.02 (0.13)	0.05 (0.29)	0.04 (0.28)	0.05 (0.23)	0.03 (0.26)	0.24 (0.62)	0.23 (0.63)	0.19 (0.56)
**19. Child ambivalence**				0.08 (0.27)	0.02 (0.13)	0.03 (0.18)	0.04 (0.19)	0.10 (0.40)	0.03 (0.18)	0.06 (0.24)	0.08 (0.34)	0.07 (0.32)	0.04 (0.20)	0.18 (0.51)	0.07 (0.26)	0.06 (0.31)	0.02 (0.13)	0.00 (0.00)
**20. Child shows fear of adult**				0.00 (0.00)	0.00 (0.00)	0.00 (0.00)	0.00 (0.00)	0.00 (0.00)	0.02 (0.13)	0.00 (0.00)	0.02 (0.13)	0.00 (0.00)	0.00 (0.00)	0.02 (0.13)	0.00 (0.00)	0.06 (0.31)	0.02 (0.13)	0.13 (0.44)
**21. Assuagement**				0.68 (0.74)	0.47 (0.60)	0.59 (0.71)	0.66 (0.72)	0.47 (0.65)	0.55 (0.71)	0.69 (0.84)	0.63 (0.69)	0.47 (0.68)	0.57 (0.67)	0.56 (0.66)	0.41 (0.62)	0.50 (0.68)	0.35 (0.58)	0.33 (0.51)
**Disorganised phenomena**																		
**22. Bizarre elements in the narrative**				**0.25** **(0.59)**	**0.03** **(0.26)**	0.22 (0.56)	0.29 (0.54)	0.15 (0.45)	0.12 (0.42)	0.25 (0.52)	0.07 (0.25)	0.10 (0.36)	0.24 (0.51)	0.13 (0.38)	0.17 (0.50)	0.22 (0.46)	0.16 (0.42)	0.17 (0.51)
**23. Child/adult gets hurt or dies**				**0.63** **(0.85)**	0.32 (0.65)	**0.26** **(0.64)**	0.15 (0.36)	0.12 (0.42)	0.16 (0.52)	0.48 (0.80)	0.22 (0.46)	0.19 (0.58)	0.23 (0.62)	0.13 (0.47)	0.16 (0.49)	0.67 (0.86)	0.61 (0.87)	0.56 (0.87)
**24. Child/adult/things are thrown away**				0.00 (0.00)	0.00 (0.00)	0.03 (0.26)	0.00 (0.00)	0.04 (0.26)	0.03 (0.26)	0.04 (0.28)	0.07 (0.37)	0.07 (0.37)	0.04 (0.28)	0.04 (0.27)	0.07 (0.37)	0.00 (0.00)	0.02 (0.13)	0.04 (0.28)
**25. Sudden shifts between good and bad**				0.00 (0.00)	0.00 (0.00)	0.02 (0.13)	0.00 (0.00)	0.04 (0.18)	0.00 (0.00)	0.00 (0.00)	0.03 (0.26)	0.00 (0.00)	0.00 (0.00)	0.00 (0.00)	0.02 (0.13)	0.00 (0.00)	0.04 (0.19)	0.12 (0.43)
**26. Sexual material**				0.00 (0.00)	0.00 (0.00)	0.00 (0.00)	0.00 (0.00)	0.00 (0.00)	0.00 (0.00)	0.00 (0.00)	0.00 (0.00)	0.00 (0.00)	0.00 (0.00)	0.00 (0.00)	0.00 (0.00)	0.00 (0.00)	0.00 (0.00)	0.02 (0.14)
**27. Disorganised behaviour**				0.22 (0.50)	0.10 (0.30)	0.16 (0.45)	0.27 (0.53)	0.14 (0.39)	0.12 (0.38)	0.19 (0.49)	0.20 (0.41)	0.14 (0.44)	0.24 (0.47)	0.27 (0.59)	0.14 (0.44)	0.06 (0.24)	0.16 (0.37)	0.10 (0.30)
**Partial score per story**				**3.33** **(2.45)**	2.42 (2.17)	**2.09** **(2.67)**	3.15 (2.47)	2.44 (2.14)	2.22 (2.22)	3.79 (2.81)	3.44 (2.82)	2.72 (1.95)	**3.57** **(2.30)**	3.14 (2.42)	**2.36** **(2.28)**	4.59 (1.98)	3.73 (2.35)	4.10 (2.77)

				**4-year-olds** N = 52				**5-year-olds** N = 59				**6–8-year-olds** N = 58				**One-way ANOVA**		
**OCTS total score** **M(SD)** **Min–max**				**3.69** (1.69) 0.20–7.50				3.06 (1.70) 0.60–8.60				**2.67** (1.80) 0.60–9.80				F(2, 166) = 4.901, p = .009 4–5 years, p = .132 5–6-8 years, p = .442 **4–6-8 years, p = .006**		

*F* = F-value, One-way ANOVA, *M* = mean, *Min* = minimum score observed, *Max* = maximum score observed, *SD* = standard deviation, *yrs* = years

Number of valid scores included in the analyses from children aged 4 years ranged between N = 48–52, between N = 54–59 for children aged 5 and between N = 56–58 for children aged 6–8

^*^The differences in scores in code 13 of the Animal stem was significant between the following groups: 4 and 5-year-olds and 4 and 6–8-year-olds

Degrees of freedom for all One-way ANOVA analyses were set to 1

The significance level is p < .05

Statistically significant age differences are highlighted in bold. All significant differences were significant at the level of p < .05

Robust test of equality of means (Brown-Forsythe) were performed in the following codes and stems: 5 (Burned Hand, Stomachache), 6 (all five conflict stems), 7 (Bike, Nightmare, Burned Hand, Stomachache), 8 (Burned Hand, Animal), 9 (all five conflict stems), 10 (Stomachache), 11 (Stomachache, Animal), 12 (Burned Hand), 13 (Bike, Nightmare, Stomachache), 14 (all five conflict stems), 15 (Bike, Stomachache), 19 (Bike, Nightmare, Stomachache), 20 (Animal), 21 (Animal), 22 (Bike, Nightmare, Burned Hand), 23 (Bike, Burned Hand), 27 (Bike, Nightmare, Stomachache, Animal), partial score of Burned Hand

#### Code Scores

Significant age differences in normative code scores were found across all age groups and in all stems except the Nightmare (see Table 5). We found the following five significant age differences between the groups of 4- and 6–8-year-olds: Bike, codes: 8 (Adult provides protection) and 23 (Child/adult gets hurt or dies), Burned hand: Code 14 (Avoidance of conflict), Stomachache: Code 9 (Adult unaware), Animal: Code 13 (Child seeks help/comfort from adult) and the following four significant age differences between the 4- and 5-year-olds: Bike: Code 22 (Bizarre elements in the narrative), Animal, codes: 8 (Adult provides help/protection), 13 (Child seeks help/comfort from adult) and 15 (Child self-care). Finally, four significant age differences were found between groups of children aged 5 and 6–8: Bike: code 7 (Adult provides comfort), Burned hand, codes: 6 (Interviewer support) and 9 (Adult unaware), Stomachache: Code 7 (Adult provides comfort). Across all the significant findings, the youngest of the age group in question consistently had higher normative code scores than the elder group. In our sample, preliminary normative code scores generally tended to decrease with increased age, however this trend did not extend to all codes across the coding scheme (e.g., normative scores in codes 2, 9, 10, 11, 12, 16, 17, 18, 20, 21, 22, 24, 25, 27, see Table 5).

In 13 code scores across the stems, all children scored 0 and one-way ANOVA could not be performed since no variation were observed (see Table 5). For 32 code scores, robust tests of equality of means (Brown-Forsythe) could not be performed because at least one of the groups had no variance. Potential age differences were thus not calculated for the following norm scores: Baseline scores: Codes 1, 4. Bike scores: Codes 1, 2, 4, 10, 11, 24, 25. Nightmare scores: Codes 2, 3, 4, 10, 11, 16, 18, 20, 25. Burned hand scores: Codes 2, 3, 11, 25. Stomachache scores: Codes 1, 3, 12, 16, 20. Animal scores: Codes 4, 12, 19, 25, 26.

### Inter-rater Reliability Analyses

#### Inter-rater Reliability Absolute Agreement

Inter-rater reliability analyses are based on double blind coding of 40–43 tests since OCTS partial and total scores assigned the value 99 were excluded from these analyses. Results are reported in Table 6. Intraclass correlation coefficients were excellent for the Burned hand and Stomachache stems, good for OCTS total and Bike stem and acceptable for the Nightmare and Animal stem.

**Table 6:** Interrater reliability analyses (N = 40–43 OCTS films)

	**Internal consistency** **Cronbach’s α [95% CI]**	**Items**	**Inter-rater reliability** **ICC [95% CI]**
OCTS total	.878 [.773, .934]	135	.869 [.750, .930]
Bike	.857 [.733, .923]	27	.855 [.732, .922]
Nightmare	.770 [.576, .876]	27	.760 [.558, .870]
Burned hand	.904 [.822, .948]	27	.902 [.820, .947]
Stomacheache	.999 [.997, .999]	27	.998 [.997, .999]
Animal	.806 [.637, .897]	27	.791 [.602, .889]

*OCTS* = Odense Child Trauma Screening, *CI* = Confidence intervals, *ICC* = Intraclass correlation coefficients

Inter-rater reliability analyses are based on double blind coding of 40–43 films since OCTS partial and total scores assigned the value 99, indicative of interviewer errors or inadequate amount of narrative material available for reliable coding, were removed from the intraclass correlation analyses

#### Internal Consistency

Cronbach’s Alpha coefficients for the OCTS total and partial scores are presented in Table 6. The Burned hand and Stomachache stems both had excellent internal consistency while the OCTS total score, Bike, and Animal stem had good internal consistency. The Nightmare stem showed an alpha value in the acceptable range.

#### Correlations Between Subscales of the OCTS, SDQ, and Trauma Exposure

For interpreting correlational values between scales of the OCTS and SDQ as well as OCTS and trauma exposure, Cohen’s guidelines (1988) were followed.

#### Correlations Depending On Sex

Correlation coefficients are shown in Table 7. Differentiating our Spearman’s rho analyses by sex, we found eight significant correlations between girls’ scores on OCTS and SDQ, while only one significant correlation was found among boys. For girls, six moderate positive correlations were found: OCTS total was associated with the following SDQ scales: Total, conduct, and hyperactivity, while scores on Bike stem was associated with scores on SDQ total, SDQ conduct, and SDQ emotional problems. A small positive correlation among girls’ scores was also found between scores on the Bike stem and SDQ hyperactivity scale. The only negative correlation between girls’ scores were found between SDQ peer problems and animal scores. The only significant correlation found for boys was a strong positive correlation between scores on the Stomachache stem and the SDQ peer problems scale. For trauma exposure, we only found a moderate positive correlation between boys’ scores on the Burned hand stem and number of traumas experienced.

**Table 7:** Spearman’s rho correlations between scores on OCTS scales, SDQ scales (n = 48–61), and trauma exposure (n = 51–61)

**Scores**	**OCTS total** **r**_**s**_**(n)**		**Bike** **r**_**s**_**(n)**		**Nightmare** **r**_**s**_**(n)**		**Burned hand** **r**_**s**_**(n)**		**Stomachache** **r**_**s**_**(n)**		**Animal** **r**_**s**_**(n)**	
	G	B	G	B	G	B	G	B	G	B	G	B
**SDQ total**	.311* (61)	-.020 (57)	.380** (61)	-.061 (57)	.226 (61)	.093 (57)	.214 (48)	-.133 (57)	.095 (60)	.206 (57)	.096 (59)	.065 (52)
**SDQ conduct problems**	.329** (61)	.038 (57)	.365** (61)	.014 (57)	.204 (61)	.175 (57)	.178 (61)	.056 (57)	.115 (60)	.019 (57)	.127 (59)	-.053 (52)
**SDQ emotional problems**	.217 (61)	-.080 (57)	.357** (61)	-.118 (57)	.166 (61)	-.029 (57)	.248 (61)	-.212 (57)	-.009 (60)	.251 (57)	-.031 (59)	.090 (52)
**SDQ hyperactivity**	.310* (61)	-.110 (57)	.266* (61)	-.072 (57)	.219 (61)	.027 (57)	.103 (61)	-.159 (57)	.188 (60)	-.025 (57)	.210 (59)	.010 (52)
**SDQ peer problems**	-.122 (61)	.092 (57)	.089 (61)	-.047 (57)	-.020 (61)	.023 (57)	-.047 (61)	-.071 (57)	-.032 (60)	.438** (57)	-.361** (59)	-.064 (52)
**SDQ prosocial**	-.246 (61)	.020 (57)	-.219 (61)	-.123 (57)	-.157 (61)	-.065 (57)	-.185 (61)	.031 (57)	-.137 (60)	.057 (57)	-.065 (59)	.124 (52)
**Trauma exposure**	.049 (61)	.198 (56)	.103 (61)	.065 (56)	.204 (61)	.134 (56)	-.111 (61)	.335* (56)	.103 (61)	.072 (56)	-.031 (59)	.184 (51)

r_s_ = Spearman’s rho correlation coefficient, *n* = number of available parent-reported SDQ-scores and reports on trauma exposure

^**^. Correlation is significant at the 0.01 level (2-tailed)

^*^. Correlation is significant at the 0.05 level (2-tailed)

#### Correlations Dependent On Age

Correlation coefficients between scales of OCTS, SDQ, and trauma exposure are shown in Table 8. Among 4-year-olds, we found significant moderate negative correlations between scores on the Nightmare stem and the SDQ prosocial subscale and between scores on the Animal stem and the SDQ peer problems scale. For 5-year-olds, five significant moderate positive correlations between scores on OCTS and SDQ scales were found: OCTS total was associated with SDQ total and SDQ peer problems, Bike scores with SDQ total, and the Nightmare scores were associated with SDQ scales for hyperactivity and peer problems. In this age group, we further found a significant moderate negative correlation between scores on the Bike stem and SDQ prosocial subscale and a significant strong positive correlation between Stomachache stem scores and the SDQ peer problems scale. For children aged 6–8, only one correlation reached significance namely a moderate negative correlation between scores on the Nightmare stem and SDQ peer problems. We only found significant correlations between OCTS scores and trauma exposure among 6–8-year-olds (OCTS total, Bike, Burned Hand).

**Table 8:** Spearman’s rho correlations between scores on the OCTS scales, SDQ scales (n = 34–45) and trauma exposure (n = 35–44)

**Scale** **scores**	**OCTS total** **r**_**s**_**(n)**			**Bike** **r**_**s**_**(n)**			**Nightmare** **r**_**s**_**(n)**			**Burned hand** **r**_**s**_**(n)**			**Stomachache** **r**_**s**_**(n)**			**Animal** **r**_**s**_**(n)**		
	4 yrs	5 yrs	6–8 yrs	4 yrs	5 yrs	6–8 yrs	4 yrs	5 yrs	6–8 yrs	4 yrs	5 yrs	6–8 yrs	4 yrs	5 yrs	6–8 yrs	4 yrs	5 yrs	6–8 yrs
**SDQ total score**	-.149 (38)	.385* (35)	.159 (45)	-.098 (38)	.353* (35)	.101 (45)	-.054 (38)	.413* (35)	.141 (45)	-.254 (38)	.183 (35)	.208 (45)	.001 (38)	.153 (34)	.251 (45)	-.116 (37)	.045 (34)	.251 (40)
**SDQ conduct problems**	-.062 (38)	.297 (35)	.181 (45)	.038 (38)	.178 (35)	.169 (45)	.060 (38)	.266 (35)	.152 (45)	-.149 (38)	.249 (35)	.171 (45)	.014 (38)	.014 (34)	.082 (45)	-.052 (37)	-.073 (34)	.229 (40)
**SDQ emotional problems**	-.257 (38)	.246 (35)	.134 (45)	-.227 (38)	.326 (35)	.155 (45)	-.102 (38)	.109 (35)	.117 (45)	-.269 (38)	.107 (35)	.180 (45)	-.062 (38)	.055 (34)	.194 (45)	-.148 (37)	-.041 (34)	.195 (40)
**SDQ hyper-activity**	.040 (38)	.207 (35)	.153 (45)	.068 (38)	.218 (35)	.053 (45)	-.023 (38)	.363* (35)	.164 (45)	-.221 (38)	.065 (35)	.141 (45)	.131 (38)	.052 (34)	.221 (45)	.060 (37)	.088 (34)	.223 (40)
**SDQ peer problems**	-.004 (38)	.433* (35)	-.258 (45)	-.093 (38)	.292 (35)	-.241 (45)	.177 (38)	.388* (35)	-.377* (45)	-.069 (38)	.140 (35)	-.158 (45)	.262 (38)	.514** (34)	-.018 (45)	-.339* (37)	.071 (34)	-.161 (40)
**SDQ prosocial score**	-.278 (38)	-.327 (35)	.117 (45)	-.136 (38)	-.357* (35)	-.043 (45)	-.375* (38)	-.172 (35)	.079 (45)	-.157 (38)	-.293 (35)	.129 (45)	-.282 (38)	.118 (34)	.121 (45)	-.092 (37)	-.054 (34)	.141 (40)
**Trauma exposure**	.071 (38)	-.079 (35)	.311* (44)	.091 (38)	-.111 (35)	.314* (44)	.177 (38)	.006 (35)	.267 (44)	-.110 (38)	-.087 (35)	.490** (44)	.062 (38)	-.015 (34)	.159 (44)	-.176 (37)	-.013 (34)	.275 (44)

*N* = number of available parent-reported SDQ-scores and reports on trauma exposure. r_s_ = spearman’s rho correlation coefficient

^**^. Correlation is significant at the 0.01 level (2-tailed)

^*^. Correlation is significant at the 0.05 level (2-tailed)

### Discussion

The aim of our cross-sectional explorative study was to generate preliminary Danish norms for the story stem screening tool Odense Child Trauma Screening (OCTS) from a diverse group of children from the general population. To the best of our knowledge, this study is the first of its kind to report normative data for a story stem tool and to do so for its entire coding scheme i.e., across all codes, partial, and total scores. While some story stem studies have broadly explored play-based and narrative responses of community or low-risk children to story stem setups as well as sex and age effects on child responses (e.g., Bretherton et al., 1990b; Del Guidice, 2008; Gloger-Tippelt & König, 2007; Green et al., 2000; Oppenheim et al., 1997; Page & Bretherton, 2003; Pierrehumbert et al., 2009; Portu-Zupirain, 2013; von Klitzing et al., 2007), our study is the first to report fine-grained data on story stem participation of a large diverse group of children. Consequently, the current study contributes to the tradition of story stem assessment and research by providing preliminary documentation for typical play and narratives across sex and ages in a diverse child group.

We found relatively few significant sex and age differences in normative code, partial, and total scores which suggest that OCTS can be applied across sexes and the age levels 4–8 years. Had many significant sex and age differences instead emerged in our sample of children from the Danish general population, results would have implied that some codes had a different pull for some of the age groups or with one sex. The lack of frequent and consistent age or sex differences across the various scores thus lends initial support to the OCTS as a tool applicable across its entire target group (children aged 4–8 years). However, the significant differences we did find point to a few areas within the coding scheme (describing certain play themes, play-behavior, or narrative representations) worthwhile of special attention in the clinical use of the OCTS. If a clinician doubts how a child’s play-based behavior and narrative representations are best understood during the scoring of a test, they can turn to the herein presented preliminary norms and conduct empirically based comparisons of the individual child’s test scores and the preliminary OCTS reference group. In such incidents, clinicians are recommended to contemplate whether the observed behavior or parts of it can be understood as an age or sex-specific phenomenon (as indicated by significantly increased scores either for the child’s age group or sex in the code describing the observed phenomenon or play-based behavior). It is, however, imperative, to stress that nuances and a certain degree of variations in children’s play-based behavior and narrative representations in story stem responses are expected to emerge within the OCTS target group (children aged 4–8) independent of the group to which a child belong (e.g., clincical group, general population) e.g., due to differences in children’s development and day-to-day functioning. This must be taken into account in the assessment in clinical practice and the preliminary norm scores must therefore be interpreted and clinically applied with caution.

With the exception of one reoccurring significant age difference between the 5- and 6- and 8-year-olds in code 7 (Adult provides comfort: Bike and Stomachache stem), none of the observed significant age and sex differences were found repeatedly across the OCTS conflict stems. It is therefore not possible to draw general conclusions about certain narrative representations or specific play-based behavior being consistently more present in the narratives of one of the sexes or age groups. Rather, the significant differences found were stem specific. What can be concluded about differences within our sample from the Danish general population is that boys, on average, scored significantly higher than girls on a few codes (n = 5) suggesting a slightly stronger effect of the pull-factor of the OCTS and its conflict stems among the sample’s boys. Further, the younger age groups had significantly higher scores than the older groups in some codes (n = 13), partial, and total scores. Significant differences in normative code scores were both found between children aged 4 and 5, 4 and 6–8 and 5 and 6–8 while significant age differences in partial and total scores (n = 3) were only found between groups of children aged 4 and 6–8. Our results thereby indicate that the OCTS partial and total scores of our sample tend to decrease with age (see Fig. 4) suggesting a somewhat weaker effect of the pull factor among the older children.

We found only few significant correlations by sex and age between scales of the OCTS and SDQ compared to the number found in the initial OCTS validity study (Løkkegaard et al., 2021). Though the difference was striking, direct comparison is impossible as Løkkegaard and colleagues (2021) investigated associations in a risk sample and conducted the analyses on the entire sample. Nevertheless, results suggest that scores on the OCTS and SDQ are not associated to the same degree in samples with children from general populations and clinical samples. This can be explained by 1) our sampling of presumably low-risk children which would entail low scores on both the OCTS and the SDQ resulting in few significant associations, 2) the instruments are developed to measure different phenomena (play-based and narrative indicators of traumatization and broader psychosocial well-being respectively), and importantly 3) informants vary between measures (OCTS: children, SDQ: caregivers). Considering these circumstances, the finding of few significant correlations is not all too surprising. Additionally, the few significant correlations suggests limited sex and age biases of the OCTS which can be considered an important test quality.

Looking further into the correlational findings, significant correlations between scales of the OCTS and the SDQ were primarily observed among girls. Interesting was the finding of three moderate correlations and one small correlation between scores on the Bike stem and subscales of SDQ (Total, conduct, emotion, hyperactivity/inattention) indicating that girls who were especially engaged in the Bike stem also presented with more caregiver-reported behavioral and emotional symptoms. Girls with higher partial Animal scores scored lower on the SDQ peer problems subscale indicative of a certain quality of the Animal stem to detect these types of psychosocial challenges in girls of our ample.

Differentiating by age, we found notably more significant correlations between scores on the OCTS and SDQ in the group of 5-year-olds (N = 8) compared to the 4- (N = 2) and 6–8-year-olds (N = 1). This difference in correlations can possibly be understood in the context of the specific developmental period the 5-year-olds of our sample were in during study participation. In Denmark, the period between the age of 5 and 6 is the common age for an institutional transition from kindergarten to school. Often, children are gradually “phased out” of kindergarten and into school by spending a certain number of hours or days during the week at the school to ease the transition. Still, the well-known everyday life of these children is dissolved as they must adjust to new physical environments, face separation from many of their kindergarten peers, make new friends, get to know, and adjust to new daily routines and rules in school. Children experience a steep increase in the demands (e.g. academic, cognitive, relational) and must establish new roles for themselves all the while fewer adult–child resources are available to them compared to kindergarten. Albeit this age and the transition from kindergarten to school is a developmentally vulnerable period possibly contributing to the higher number of significant correlations identified in this age group.

Lastly, we only found significant positive correlations between trauma exposure and scores on the OCTS in the sample’s oldest group of children indicating that as trauma exposure increased in children aged 6–8, so did their scores on the OCTS total, Bike and Burned hand stem. OCTS being a trauma screening tool, one could expect to find more significant associations, however, as reported by caregivers, a large proportion of the children in our study (61.5%) had not previously been exposed to any of the potentially traumatic events on the DIPA list why such possible associations between OCTS scores and trauma exposure would not be possible to detect. This could contribute to explain the few significant associations found between OCTS scores and trauma exposure.

### Methodological Considerations

Several study limitations must be taken into account for the interpretation and utilization of the preliminary Danish OCTS norms. Importantly, we did not assess or control for children’s language abilities as some previous story stem studies have done. To mention a few of them, some studies have reported findings where significant differences in children’s story stem results depending on variables such as group-membership and sex became non-significant when language competencies were controlled for (Stievenart et al., 2014; von Klitzing et al., 2007). Other studies have found no such significant effect of language abilities on story stem results such as secure representations (Shin, 2019) or attachment classification (Granot & Mayseless, 2012). The current evidence on the potential effect of language abilities on a range of story stem outcomes therefore seems ambiguous, and as we did not explore a potential mechanism between OCTS results and language abilities, we cannot deduce if or how language abilities of the included children might have affected their OCTS results and the reported preliminary norms.

In addition to language abilities, a wide array of other developmental, contextual, relational, and social factors might influence children’s prerequisites for engaging in narrative play observations (Allen et al., 2018; Dealy et al., 2017; Short et al., 2011; Tang et al., 2018). Cognitive, neurological, cultural, and sensory factors as well as current child life stressors, ability to pretend play, assessment context, child-interviewer interaction, and child interest in and familiarity with play materials might all play crucial, potential interactional, roles in influencing children’s story stem responses. As we did not assess or control for any of these factors, some or several might have exerted influenced on participating children’s OCTS responses and the preliminary norms without us being able to investigate and document the potential effects. This is an important limitation to consider when applying the preliminary OCTS norms in clinical practice or in research settings. Especially so in light of previous story stem studies documenting associations between certain play behavior (e.g., disorganization), poorer arousal modulation, narrative coherence as well as atypical themes (e.g., role reversal, bizarre content, injury) and symptoms of child psychopathology (e.g., mood disturbances: Beresford et al., 2007; Hutchison et al., 2010; Luby et al., 2009), behavior problems: Wan & Green, 2010), anxiety (Warren et al., 2000), attachment disorders (RAD: Minnis et al., 2009) and developmental disorders (ADHD: Green et al., 2007)). Obtaining more nuanced and richer data about recruited children’s developing cognitive, emotional, relational, and language skills must remain a priority for future studies with the OCTS. If feasible, this would allow for investigation and documentation of potential associations between developmental factors on children’s play-based behavior and narrative responses to the OCTS. Furthermore, this would strengthen diagnostic differentiation and interpretation of test results and thereby contribute to more valid and reliable interpretation of OCTS test results.

The generalizability of the study’s results is limited because the sample primarily was recruited from one region of Denmark, and children were signed up for participation by caregivers. The sample was therefore not representative but one of availability. It was presumably skewed because caregivers with more resources were more likely to sign them and their child up for participation e.g., participation was voluntary and not compensated in any way, the study information was comprehensive and given electronically through many documents. When utilizing the preliminary normative scores, attention as to how an individual child might differ demographically from the study sample is therefore warranted.

The reported results for the group of children aged 6–8 are also limited since we, despite considerable recruitment efforts, did not succeed in recruiting the intended number of children across the ages 6, 7, and 8. Possible reasons for this could be that the data collection was conducted during the COVID-19 pandemic characterized by changing societal guidelines and restrictions imposed by the Danish Health Authorities. Across all school personnel, extensive resources were therefore spent on trying to keep up with and adhering to the changing guidelines in addition to upholding normal teaching, pedagogical, and administrative activities. This was especially true in the first part of the data collection where very few schools responded to our invitation. When restrictions were eased in the second part of the data collection, many schools still wished to limit participation in external projects since they were now heavily preoccupied with trying to return to their pre-pandemic procedures and routines. We therefore do not know whether additional significant differences in norm code, partial, or total scores would have emerged between sexes or age groups or if the current identified differences would still hold, had we been able to recruit the intended number of children aged 6–8. With the sampling of a smaller non-representative group, our study thus needs to be replicated with larger representative samples.

Though the inter-rater reliability and internal consistency ranged from excellent to acceptable for all partial and total scores, the preliminary normative scores might also be influenced by the coders’ educational level and lack of clinical experience. As coders were all psychology students in the bachelor or master’s psychology program at University of Southern Denmark, few had had the chance to gain clinical experience e.g., with psychological child assessment and identification of disorganized phenomena and behavior. We sought to compensate for this by conducting an extensive amount of training and supervision throughout the coding processes and by examining inter-rater reliability at the level of partial and total scores. However, we did not examine inter-rater reliability at code level, nor did we investigate intra-rater agreement, a measure of a coder’s consistency in repeated scoring of phenomena (Alavi et al., 2022). Doing so could have informed us further of individual coding abilities or tendencies with regards to identifying and coding the play-based behavior, phenomena, and narrative representations contained in the OCTS coding system thus increasing the confidence in the reliability of the codings and reported normative scores. If feasible, including and assigning coders with a higher degree of clinical experience i.e., researchers, certified OCTS administrators and coders and clinical pediatric psychologists as a priori coders for double blind coding in future studies with the OCTS would increase both the validity and reliability of scores.

## Conclusion

Preliminary Danish norms for the entire coding system of the OCTS were established from a large diverse group of children aged 4, 5 and 6–8 years from the general population. Sex and age differences in play-based behavior and narrative representations were investigated across code, partial and total scores. No significant sex differences were detected in OCTS partial scores or total score. Significant sex differences in normative code scores were only found in the narrative responses to three of the five OCTS stems with boys scoring higher in all instances (more bizarre elements in the narrative of boys in response to the Nightmare stem, child protagonist displayed more self-help and more disorganized phenomenon were present in the Stomachache narrative of boys, and adult representations displayed as more aggressive and less helpful/comforting among boys in Animal narratives).

Sixteen significant age differences were found across all conflict stems, except the Nightmare stem, and across all score levels (code, partial, and total scores): Eight were found between the groups of 4 and 6–8-year-olds while four were found both between the 4- and 5-year-olds and between the 5- and 6–8-year-olds. Contrary to differences depending on sex, significant age differences were found in normative partial scores (Bike and Stomachache stem) and in the OCTS total score with differences only emerging between the 4- and 6–8-year-olds. For all significant age differences, the youngest of the two groups in question presented with higher norm scores. These relatively few age and sex differences in the OCTS stories and coding scheme lend preliminary support to the OCTS as a tool applicable for utilization across a diverse group of children within the target group of children aged 4–8 years.

The preliminary normative scores will prove especially valuable in the OCTS assessment of children with ambiguous test results and where doubt arises as to how the child’s play-based behavior and narrative representations is best scored and understood. In such incidents, normative data provide information about the standing of a child relative to the reference group enabling psychologists to better distinguish between clinically relevant OCTS test results, where cause for further assessment arises, and test results within a preliminary normative range.

Future studies should aim to generate more normative data for the remaining part of the OCTS target group (children aged 6–8) or ideally focus on recruiting larger representative samples to extract full norms from. This would increase the strength of data, the psychometric qualities of the OCTS, generalizability and further benefit the clinical assessment of children with the OCTS. As all story stem-based assessment benefits psychometrically from having access to a reference group (norms), we hope that our study can inspire fellow researchers within the story stem field to establish norms for other existing story stem tools. Studies exploring potential similarities or differences in play-based behavior and narrative representations across different groups of children (e.g., children with developmental disorder or other clinical groups) and investigating how a variety of factors might influence children’s participation in story stems (e.g., cognition, language abilities, trauma exposure, culture) are also warranted.

## Supplementary Information

Below is the link to the electronic supplementary material.

Online resource 1 (PDF 172 KB)
