# Supplementary material for: Preliminary Danish Norms for the Odense Child Trauma Screening (OCTS)
Source: J Child Adolesc Trauma. 2024 Apr 4;17(3):805–29. doi: 10.1007/s40653-024-00616-7 (PMC11413271; doi:10.1007/s40653-024-00616-7)
Supplement: Supplementary file 1 — Online resource 1 (PDF 172 KB) [file 40653_2024_616_MOESM1_ESM.pdf]

Online Resource 1: OCTS Coding scheme

	Base-line	Story stem 2 Bikes	Story stem 3 Night-mare	Story stem 4 Burnt hand	Story stem 5 Stomach ache	Optional Animal story stem
**Engagement and narrative production**	**Score**	**Score**	**Score**	**Score**	**Score**	**Score**
1. Engagement						
2. Arousal						
3. Arousal modulation						
4. Ability to keep within task boundaries						
**Nature of the narrative**	**Score**	**Score**	**Score**	**Score**	**Score**	**Score**
5. Narrative coherence						
6. Interviewer support						
**Adult representations in the narrative**	**Score**	**Score**	**Score**	**Score**	**Score**	**Score**
7. Adult provides comfort						
8. Adult provides help/protection						
9. Adult unaware						
10. Adult rejects child						
11. Adult shows aggression						
12. Adult is controlling						
**Child representations in the narrative**	**Score**	**Score**	**Score**	**Score**	**Score**	**Score**
13. Child seeks help/comfort from adult						
14. Avoidance of conflict						
15. Child self-care						
16. Child ‘parents’ or ‘controls’ adult						
17. Excessive compliance in child						
18. Child shows aggression						
19. Child ambivalence						
20. Child shows fear of adult						
21. Assuagement						
**Disorganised phenomena**	**Score**	**Score**	**Score**	**Score**	**Score**	**Score**
22. Bizarre elements in the narrative						
23. Child/adult gets hurt or dies						
24. Child/adult/things are thrown away						
25. Sudden shifts between good and bad						
26. Sexual material						
27. Disorganised behaviour						
Partly score per story						
**Total score**						

Recoding of weighted scores: Red: Scores of 1 or 2 = **1;** Yellow: Score 1 = **0**, score 2 = **1;** Green: Scores of 0, 1, or 2 = **0;** Not codable = 99
